# Quantifying Irrigated Winter Wheat LAI in Argentina Using Multiple Sentinel-1 Incidence Angles

**DOI:** 10.3390/rs14225867

**Published:** 2022-11-19

**Authors:** Gabriel Caballero, Alejandro Pezzola, Cristina Winschel, Alejandra Casella, Paolo Sanchez Angonova, Luciano Orden, Katja Berger, Jochem Verrelst, Jesús Delegido

**Affiliations:** 1 Agri-Environmental Engineering, Technological University of Uruguay (UTEC), Av. Italia 6201, 11500 Montevideo, Uruguay; 2 Image Processing Laboratory (IPL), University of Valencia, C/Catedrático José Beltrán 2, Paterna, 46980 Valencia, Spain; 3 Remote Sensing and SIG Laboratory, Hilario Ascasubi Agricultural Experimental Station, National Institute of Agricultural Technology (INTA), Hilario Ascasubi 8142, Argentina; 4 Permanent Observatory of Agro-Ecosystems, Climate and Water Institute-National Agricultural Research Centre (ICyA-CNIA), National Institute of Agricultural Technology (INTA), Nicolás Repetto s/n, Hurlingham, Buenos Aires 1686, Argentina; 5 Centro de Investigación e Innovación Agroalimentaria y Agroambiental (CIAGRO-UMH), GIAAMA Research Group, Universidad Miguel Hernández, Carretera de Beniel Km, 03312 Orihuela, Spain; 6 Mantle Labs GmbH, Grünentorgasse 19/4, 1090 Vienna, Austria

**Keywords:** leaf area index, Sentinel-1, time-series, local incidence angle, Whittaker smoother, Gaussian processes regression

## Abstract

Synthetic aperture radar (SAR) data provides an appealing opportunity for all-weather day or night Earth surface monitoring. The European constellation Sentinel-1 (S1) consisting of S1-A and S1-B satellites offers a suitable revisit time and spatial resolution for the observation of croplands from space. The C-band radar backscatter is sensitive to vegetation structure changes and phenology as well as soil moisture and roughness. It also varies depending on the local incidence angle (LIA) of the SAR acquisition’s geometry. The LIA backscatter dependency could therefore be exploited to improve the retrieval of the crop biophysical variables. The availability of S1 radar time-series data at distinct observation angles holds the feasibility to retrieve leaf area index (LAI) evolution considering spatiotemporal coverage of intensively cultivated areas. Accordingly, this research presents a workflow merging multi-date S1 smoothed data acquired at distinct LIA with a Gaussian processes regression (GPR) and a cross-validation (CV) strategy to estimate cropland LAI of irrigated winter wheat. The GPR-S1-LAI model was tested against in situ data of the 2020 winter wheat campaign in the irrigated valley of Colorador river, South of Buenos Aires Province, Argentina. We achieved adequate validation results for LAI with ${\text{R}}_{CV}^{2}=0.67$ and RMSE_*CV*_ = 0.88 m^2^ m^−2^. The trained model was further applied to a series of S1 stacked images, generating temporal LAI maps that well reflect the crop growth cycle. The robustness of the retrieval workflow is supported by the associated uncertainties along with the obtained maps. We found that processing S1 smoothed imagery with distinct acquisition geometries permits accurate radar-based LAI modeling throughout large irrigated areas and in consequence can support agricultural management practices in cloudprone agri-environments.

## 1 Introduction

Remote sensing synthetic aperture radar (SAR) satellites have ample potential for the monitoring of vegetation biophysical variables [1–3]. Radar, as a valuable Earth observation (EO) source, allows all-weather image acquisition even during day or night time. In most tropical and cloudy regions, the usage of optical satellite imagery is often restricted to cloud-free acquired scenes typically related to a dry season [4]. This condition makes SAR data attractive for year-round cropland biophysical variable retrieval [5]. In recent years, the exponential evolution of EO as an applied science has generated an unprecedented amount of research using SAR satellite data for crop trait monitoring over large areas, e.g., [6–12]. At the field scale, various studies have employed radar backscatter to find a relationship between the electromagnetic signal and vegetation dynamics [7]. Ferrazzoli et al. [13] found a strong correlation between C-band HV-polarized backscatter and the biomass of colza, wheat, and alfalfa crops (R^2^ = 0.75). Frequently, cross-polarized (CP) radar backscatter is most sensitive to volume scattering mechanisms such as those produced by the vegetation. High correlations have been found between C-Band CP backscatter and crop biophysical variables such as leaf area index (LAI) and biomass [13,14].

The radar backscatter signal is influenced by cropland biomass and the three-dimensional structure of vegetation [15] and also by the ground soil moisture and roughness [16]. The radar acquisition geometry configurations used for the observations play a significant role [17–19]. Regarding vegetation trait retrieval based on radar signals, monitoring of wheat croplands deserves special attention. Multiple studies have explored the backscatter-vegetation structure interaction for winter wheat crops [20–25]. There is consensus that the vegetation backscatter merges the contribution of the soil-canopy interaction (surface scattering) with the backscatter from the canopy layer (volumetric scattering). Regarding wheat plant structure, the following dominant backscatter sources can be distinguished: stem, leaves, and ears [20]. The wheat phenological stage plays a significant role in the vegetation scattering detected by the spaceborne radar instruments [26].

SAR time-series data has been used to track the complete phenological cycle of summer (sunflower, maize, and soybean) and winter (barley, rapeseed, and wheat) crops [27]. SAR imagery for cropland monitoring strongly depends on the radar backscatter incidence angle, thus, it is one of the most critical obstacles [28]. The local incidence angle (LIA) is depicted by the incident radar beam and the normal to the surface, considering local relief, typically derived from a digital elevation model (DEM) [29]. When a homogeneous crop paddock is monitored regularly using radar data, the variability of the backscatter can be attributed, among others, to the LIA of the acquired radar scenes. LIA values between 35° and 40° maximize the vegetation’s volumetric scattering increasing the path length of the radar signal [30]. At the other extreme, incidence angles lower than 30° increase the ground surface scattering related mainly to soil moisture and roughness [31]. For scenes observed at shallow incidence angles, the backscatter is lower than those acquired at steeper incidence angles; in consequence, the same backscatter element in a radar resolution cell has distinct and incomparable values of backscatter coefficient for distinct local incidence angles of observation [32,33]. Accounting for angularity, Kaplan et al. [22] studied two transformations that allow the collective usage of Sentinel-1 (S1) imagery for agricultural monitoring of wheat, tomatoes, and cotton in Israel. They normalized the local incidence angle to improve the empirical prediction of LAI.

S1 is a C-band SAR satellite constellation of the Copernicus program belonging to the European Space Agency (ESA). As the pre-defined observation strategy over land, S1 defines the interferometric wide swath (IW) mode. This mode is available for most of the applications in dual-polarization (VV and VH), at 10 × 10 m spatial resolution in a swath of 250 km [34]. S1 mission consisting of S1-A and S1-B satellites provides exhaustive monitoring of Earth’s surface once every 6 days at the equator, in a single pass (ascending or descending) for one specific relative orbit. In intensive agriculture sites where several S1 relative orbits lead to multiple-path radar observations, dense time-series of sundry acquisition geometries can be obtained for the same cropland. Vegetation’s structure geometry, as a proxy of the radar backscatter, changes as a function of the local incidence angle and plants’ growth [16].

The S1 polarimetric bands VH and VV at the C-band provide relevant information on surface scattering (VV polarization) and vegetation volumetric scattering (VH polarization), which allows monitoring of soil moisture and roughness as well as vegetation biophysical parameters. The relationship between the S1 polarimetric bands and the vegetation traits can be linear or nonlinear. Harfenmeister et al. [35] applied linear and exponential regressions to assess the correlation between absolute and relative vegetation water content, LAI and plant height, and the S1 VH + VV bands. The nonlinear relationship between the S1 polarimetric data and vegetation properties, such as LAI, can be learned by machine learning regression algorithms (MLRA). Considering this strategy, in the last few decades, a variety of MLRAs have been auspiciously applied for recovery tasks mainly on optical data, e.g., decision trees, artificial neural networks, or kernel-based methods [36,37]. Gaussian process regression (GPR) [38] arose as a advantageous kernel-based non-parametric regression and has been broadly utilized in studies retrieving vegetation’s traits from EO data [39–47]. GPR as probabilistic MLRA also provides the uncertainty of the estimates making it an appealing option for vegetation trait confidence mapping.

Research in time-series remote sensing data is receiving large attention. The construction of high spatio-temporal data cubes derived from multi-temporal EO images requires the implementation of a data smoothing process to facilitate the processing and analysis of time-series [48]. The Whittaker smoother emerged as an attractive algorithm for filtering EO data in the presence of data gaps [49]. The algorithm is easy and intuitive to use, often gives superior results faster than the widely used Savitzky–Golay smoother [50].

When it comes to the viability of generating vegetation trait maps from S1 time-series data, several experimental studies made use of the polarimetric bands considering the same relative orbit [17,51]. However, the backscatter variation at distinct local incidence angles for crop biophysical variables monitoring has been left largely unexploited. Therefore, and with the ambition to improve vegetation trait retrieval, this research aims to assess the potential of combining S1-A and S1-B C-band high-resolution SAR data acquired at distinct local incidence angles. To assess the possibilities of continuously monitoring cropland’s traits with a space-based strategy, this study carries out the development of an S1-based LAI retrieval workflow devoted to irrigated winter wheat cropland monitoring in Argentina. Given the above-sketched general framework, the following three sub-objectives can be defined: (1) to develop a GPR model for an explicit quantification of winter wheat LAI from S1-A and S1-B smoothed data acquired at multiple local incidence angles; (2) to generate accurate S1-based maps of the wheat LAI with the inclusion of associated uncertainties over an intensive irrigated agroecosystem; and (3) to evaluate LAI time-series identifying phenological stages over the selected study site.

## 2 Materials and Methods

### 2.1 Theoretical Background

Aiming to describe the theoretical framework implemented in our research, this section presents the mathematical formulations of the Gaussian processes regression algorithm (Section 2.1.1) and the optimized Whittaker smoother (Section 2.1.2).

#### 2.1.1 Gaussian Processes Regression

During the last decade, GPR has been widely adopted for bio-geo science applications [52]. GPR generalizes the Gaussian probability distribution in a function’s space. This means an expected covariance between values of the function at a given point [38]. In short, a GPR model establishes a relation between the input (here: S1 polarimetric bands) *x* ∈ R^𝔹^ and the output variable (vegetation’s biophysical variable to be retrieved) *y* ∈ ℝ. Being *x*= [*x*^1^,…, *x^B^*] ∈ ℝ^*B*^ the S1 polarimetric training dataset and *y* ∈ ℝ the model’s output estimates, the mean expected value *ŷ* for a given x_*_ can be expressed in the form:

(1) $$\hat{y_{*}}=f\left(x_{*}\right)=\overset{N}{\underset{i=1}{\sum^{\text{​}}}}\alpha_{i}k_{\theta }\left(x_{i},x_{*}\right)+\alpha_{0},$$

where ${\left\{x_{i}\right\}}_{i=1}^{N}$ are the S1 polarimetric features used in the learning (or training) phase, *α_i_* ∈ ℝ is the weight assigned to each one of them, *α*_0_ a constant value (bias) of the regression function, and *k*_θ_ is a covariance function (or kernel) assessing the similarity between thetest S1 polarimetric data x_*_ and all *N* *x_i_* in the training dataset. The regression kernel is parameterized by a set of parameters *θ* = [*v*, *σ_n_*, *σ*_1_, … *σ_B_*].

The core of GPR as a kernel method relies on a convenient definition of the covariance function. We implemented automatic relevance determination (ARD) as a kernel function *k_θ_*, which can be noted as:

(2) $$k\left(x_{i},x_{j}\right)=v\exp\left(-\overset{B}{\underset{b=1}{\sum^{\text{​}}}}\frac{{\left(x_{i}^{b}-x_{j}^{b}\right)}^{2}}{2\sigma_{h}^{2}}\right)+\sigma_{n}^{2}\delta_{ij},$$

where $x_{i}^{b}$ represents the feature f of the input vector *x_i_*, v is a scaling factor, *σ_b_* is the standard deviation of the observations assumed to be noisy, *σ_f_* is the length scale per S1 input feature (*b*= 1,..., *B*), and *δ_i_j* is the correlation length scale parameter.

ARD is a widely used covariance function that typically suffices to address most of the remote-sensing-based applications [39]. The weights *α_i_* are obtained by an automatic optimization process. The 1/*σ_b_* values represent the relevance of the S1 polarimetric feature used to train the GPR model [53,54].

#### 2.1.2 Optimized Whittaker Smoother

Here, we present the Whittaker [55] smoother and interpolator mathematical theory. Whittaker smoother is usually regarded as an example of a non-parametric regression algorithm [56]. He proposed solving a regularized least-squares problem in which a scalar parameter determines the trade-off between fidelity to the data and smoothness of the filtered sequence [57].

Given a sequence of n measurements {*y*_1_, *y*_2_, …, *y_n_*}, a positive real number *λ*, and a positive integer *p* < *n*, find the sequence {*x*_1_, *x*_2_, …, *x_n_*} that minimizes:

(3) $$\lambda \overset{n}{\underset{j=1}{\sum^{\text{​}}}}{\left(y_{j}-x_{j}\right)}^{2}+\overset{n-p}{\underset{j=1}{\sum^{\text{​}}}}{\left({\text{Δ}}^{p}x_{j}\right)}^{2}$$

where Δ is the forward difference operator:

(4) $$\text{Δ}x_{j}=x_{j+1}-x_{j}$$

(5) $${\text{Δ}}^{2}x_{j}=\text{Δ}\left(\text{Δ}x_{j}\right)=x_{j+2}-2x_{j+1}+x_{j}$$

The first term in the sum in Equation (3) measures fidelity to the data, and the second measures smoothness, in that sense, the polynomial of degree *p* – 1 means maximal smoothness. The parameter *λ* determines the strength of the smoothing. General applications require that *λ* needs to be tuned over several orders of magnitude (10, 100, 1000). Optimal values of *λ* should provide a smooth curve that reveals the true nature of the data whilst removing roughness and randomness [58].

Let y^*T*^ and x^*T*^ be of the form:

(6) $$y^{T}=\left[y_{1}\;y_{2}\;\cdots \;y_{n}\right]$$

(7) $$x^{T}=\left[x_{1}\;x_{2}\;\cdots \;x_{n}\right]$$

The objective function in Equation (3) can be written as:

(8) $$\lambda (y-x{)}^{T}(y-x)+x^{T}M^{T}Mx$$

where *M* is a (*n* – *p*) *xn* differencing matrix. Given *p* = 2 and *n* = 6, the resulting *M* matrix looks like follows:

(9) $$M=\left(\begin{array}{cccccc} 1 & -2 & 1 & 0 & 0 & 0\\ 0 & 1 & -2 & 1 & 0 & 0\\ 0 & 0 & 1 & -2 & 1 & 0\\ 0 & 0 & 0 & 1 & -2 & 1 \end{array}\right)$$

The minimizer of Equation (8) is the solution of the normal equations:

(10) $$A\hat{x}=\lambda y$$

where

(11) $$A=\lambda I+M^{T}M$$

The solution can be obtained via:

(12) $$\hat{x}={\left(I+\lambda^{-1}M^{T}M\right)}^{-1}y$$

or,

(13) $$\hat{x}=y-\lambda^{-1}M^{T}M\hat{x}$$

As an optional second parameter, the default second-order smoother (*p* = 2) will work fine for almost all signals, and the first p moments of the data are preserved. One popular approach to solve Equation (13) is to perform a search for an optimal criterion over a fine grid of *λ* values seeking to minimize the loss function. The use of generalized cross-validation (GCV) [59] emerges as an appealing alternative to deal with this issue. With this automatic method of choosing the smoothing parameter, *λ* is selected to minimize the GCV score:

(14) $$n^{-1}\overset{n}{\underset{j=1}{\sum^{\text{​}}}}{\left(\frac{y_{j}-{\hat{x}}_{j}}{1-n^{-1}trace\left(\lambda A^{-1}\right)}\right)}^{2}$$

Cross-validation aims to optimize the predictions of miss-out sample data. The prediction accuracy increases when the correlation between neighbor samples is exploited rather than utilizing the values of a smoothed trend [49]. The V-curve was proposed by Frasso and Eilers [60] as a tool to explore the smoothing parameter space. It is a modification on Hutchinson and Hoog [61] L-curve, which is a plot of (*ψ*,*ϕ*), evaluated on a fine grid of log *λ*, with:

(15) $$\psi =\log\underset{j}{\sum^{\text{​}}}\omega_{i}{\left(y_{j}-{\hat{x}}_{j}\right)}^{2},\phi =\log\underset{j}{\sum^{\text{​}}}{\left({\text{Δ}}_{x_{j}}^{p}\right)}^{2}$$

The Whittaker smoother combined with the V-curve for the optimal selection of the smoothing parameter *λ*, is a simple, fast, and powerful tool for EO data processing [49].

### 2.2 Study Site

**Figure 1:**
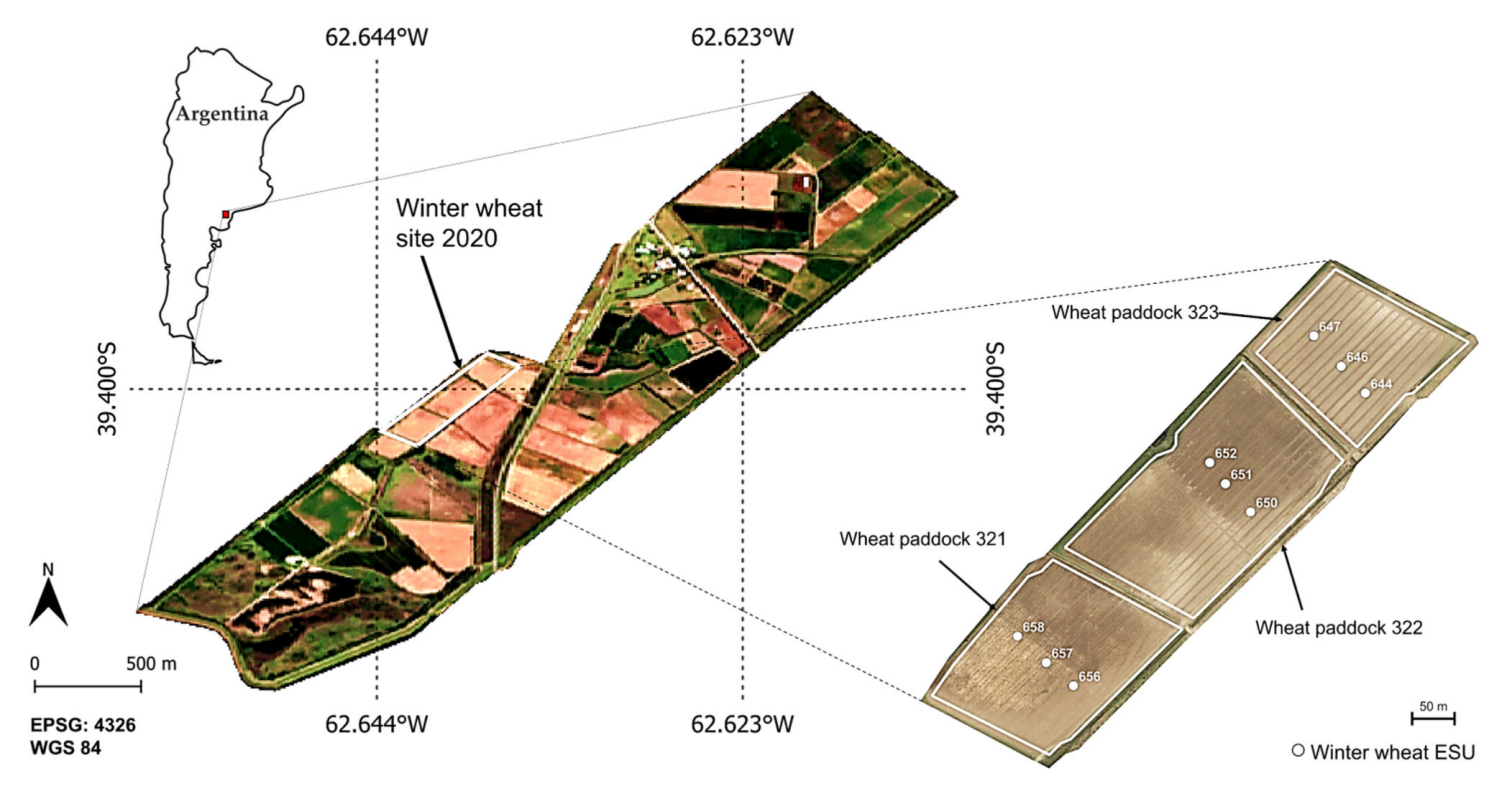
Test fields of irrigated winter wheat in the study site. True color S2 image of 27 December 2020, partly adapted from [63]. Reference system: WGS84 (EPSG 4326).

The study site is located in the South of Buenos Aires Province, Argentina. The Bonaerense valley of the Colorado river (BVCR) is an intensively cultivated and irrigated Agri-environment occupying 91,163 ha, that includes horticulture, pastures, and cereals [62]. Most of the agricultural practices in the study region have been possible thanks to gravity irrigation [63]. To address our study, we selected three winter wheat paddocks belonging to the 2020 crop campaign in the BVCR (see Figure 1).

#### 2.2.1 Characteristics and Environment of the Study Region

The Buenos Aires Province’s southern segment made up of the Villarino and Patagones districts, has a temperate Mediterranean arid steppe climate according to the Köppen’s climate classification [64]. The landscape presents aridity features in the vicinity of the Río Negro Province. Rainfall is greatest in autumn and decreases from the north to the south-west, with 483.5 mm on average. The estimated annual water deficit in the irrigated area is 322 mm on average, while in the extreme South of the territory it exceeds 400 mm [65]. The rainfall regime alternates between periods with water abundance and extreme droughts. The average annual temperature is 14.8 °C, with the lowest records of 1.6 °C registered in July and the highest values at around 30 °C for January. The annual frost-free period is more than 240 days in the East of the territory.

In the BVCR irrigated area, cropland productivity is affected by two soil issues. Primarily, soil salinity is related to low drainage capacity and soil cutting, which is associated with surface conditioning labors performed to obtain optimal water drainage. In general, the soils are Entic Hapludoll with sandy-to-sandy loam texture according to the USDA Soil Taxonomy classification [66], with levels of organic matter close to 2.3 % [67]. Due to its texture, water retention is low, and the risk of erosion is high.

#### 2.2.2 Winter Wheat Cropland Development and Properties in the BVCR

When periods of drought occur in the initial stages of wheat development, lack of crop uniformity is usually observed, attributed to the physio-chemical composition of the soil [68]. In the middle of the booting stage, the color of the cropland is generally darker in the less soil-worn sectors. Wheat croplands require low salinity content soils (<4 dS m^−1^), neutral pH, high phosphorus concentration (>20 ppm P-soluble), and a good level of organic matter (>1.5 %) [69]. The soil must be prepared for the conduction of water, usually under the modality of planks situated every 10 to 14 m [69]. Long-term cycle wheat can be sowed by the end of May, whereas this condition can be extended to the middle of August for short-term cycle wheat [69]. In all cases, it is recommended to take care that the soil profile is recharged with moisture in the planting stage. It is recommended to adjust the seed dose to obtain between 250 and 350 plants m^−2^ during the sowing time. If the profile was effectively recharged for the planting period, the wheat meets the tillering stage without suffering water deficit. After that, at the beginning of the jointing (stem elongation), it is recommended to carry out irrigation when the first visible knot appears, another in pre-flowering (booting), and the third when the heading of the crop has finished.

### 2.3 Field Data Collection for Training and Validation

Three winter wheat paddocks were sown simultaneously on 25 June 2020. Fertilization was applied in two instances throughout the plants’ tillering stage, first by the end of August 2020 and second by the middle of September with 200 kg ha^−1^ of nitrogen (Urea: 46-0-0) manually uniformly distributed. Three gravity irrigations were performed throughout the crop cycle during late August, middle of September, and late November. A comprehensive overview of the BVCR’s wheat campaign 2020 and an exhaustive description of the cropland management and experimental design are provided by Caballero et al. [63]. A total of 9 Elementary Sampling Units (ESU) were visited periodically during the wheat development periods from August to December. Each ESU was restricted to a 10 × 10 m area, analogous to the pixel size of the post-processed S1 imagery.

**Figure 2:**
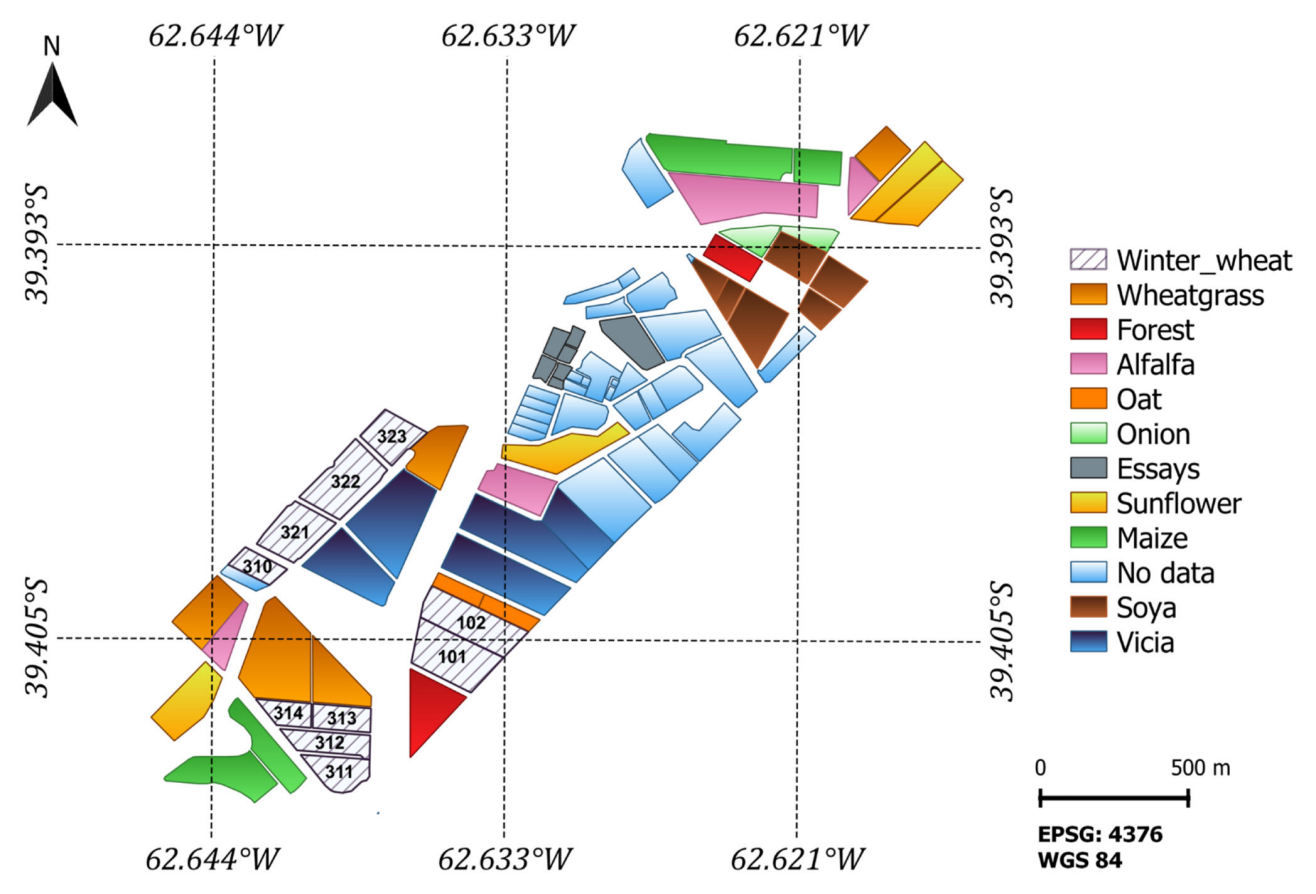
Study site 2020 land use. Reference system: WGS84 (EPSG 4326).

LAI samples were acquired utilizing the PocketLAI R Smart-App [70]. Six observations (n = 6) were performed per ESU and then averaged to boost statistics robustness (See Table 1). Winter wheat phenology was established regarding secondary growth stages defined by the Zadoks-scale [71] and registered in the in situ 2020 database. Winter wheat field data and land use were collected by a group of professionals at the Hilario Ascasubi Experimental Station (HAEE) belonging to the National Institute of Agricultural Technology (INTA), Argentina. The HAEE’s land use for the 2020 cropland campaign is displayed in Figure 2.

#### 2.3.1 Winter Wheat Phenology and Meteorological Data Trend

**Figure 3:**
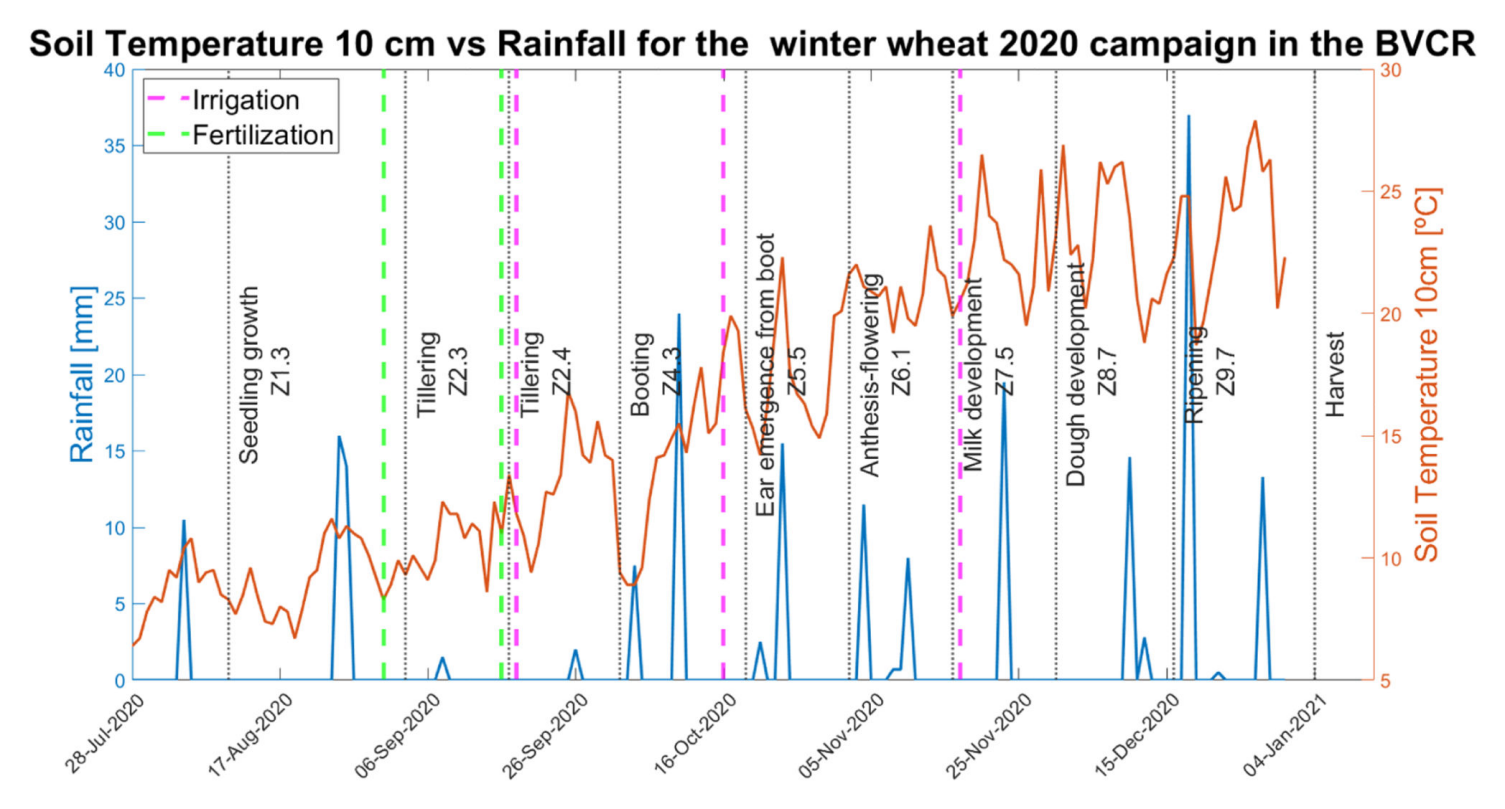
Meteorological data, soil temperature at 10 cm, and rainfall in the BVCR study site against winter wheat phenological stages.

The meteorological data were supplied by the INTA-Hilario Ascasubi weather station situated in the study site “39.38°N, 62.62°W” (Villarino district - Buenos Aires Province) very close to the wheat crop paddocks selected for this study. Available meteorological stations can be queried through the following website: http://siga.inta.gob.ar/#/(accessed on 19 September 2022). A time-series consisting of daily soil temperature at 10 cm (°C) and precipitation (mm) data ranging from the 1 September 2020 to 31 December 2020 were downloaded and analyzed. Figure 3, shows the wheat plant phenological stages evolution (described by the Zadoks-scale), irrigation and fertilization moments, and the meteorological data trend.

### 2.4 Sentinel-1 SAR Data Processing

An automatic-processing chain was developed using the Sentinel-1 Toolbox provided by SNAP (Sentinel Application Platform) software version 8.0 https://step.esa.int/main/snap-8-0-released/(accessed on 20 September 2022). We downloaded from ESA’s website https://scihub.copernicus.eu/(accessed on 19 September 2022), a total of 35 S1-A and S1-B images between 27 August 2020 to 12 January 2021 consisting of Ground Range Detected (GRD) IW products in dual polarization VV+VH, providing a 6-day revisited time over the study site. The selected relative orbit numbers (RON) or S1 paths were 141 for S1-A and 68 for S1-A and S1-B both orbits are descendent. The S1 acquisition time was around 6:20 h local time. The local incidence angle over the winter wheat paddocks varies from roughly 30° to 37° for path 141 and from 40° to 47° for path 68. First, the S1 precise orbits were applied to all S1 images followed by a thermal noise-removing process. Subsequently, the S1 scenes were calibrated radiometrically obtaining the normalized backscatter coefficient *γ*_0_ (Gamma Naught). Afterward, the range doppler terrain correction was utilized to geocode the images precisely using the Shuttle Radar Topography Mission (SRTM) high-resolution DEM. The resulting spatial resolution for the S1 IW-GRD product without multi-looking was set to 10 × 10 m. Following this, the pre-processed S1 images were filtered to minimize the speckle effect. We selected the IDAN filter (Intensity-Driven-Adaptive-Neighborhood) [72] with the following filter configuration parameters: Adaptive Neighbor Size = 5, Number of Looks = 2. Finally, the S1 pre-processed images were spatially filtered by applying a 3 × 3 mean filter and restricted to the study site.

Considering that the S1 images have distinct observation paths, a sub-pixel accurate fine coregistration process is mandatory to ensure that each ground target contributes to the same (range, azimuth) pixel in all the S1 observed scenes [73]. We employed the following fine coregistration configuration parameters: Resampling type = None, Initial offset method = Orbit, Number off ground control points = 2000, RMS threshold (pixel accuracy) = 0.05, Warp polynomial order = 1, Interpolation method = Cubic convolution (6 points). Table 2 shows the correspondence between in situ sampling dates and S1 acquisitions.

### 2.5 Sentinel-1 Time-Series Smoothing and interpolation

The Whittaker smoother for time-series interpolation was implemented in Python version 3.6.13 (https://www.python.org/downloads/release/python-3613/, accessed on 22 September 2022) utilizing the vam.whittaker version 2.0.2 (https://github.com/WFP-VAM/vam.whittaker, accessed on 22 September 2022) core functionality used in the MODIS Assimilation and Processing Engine (MODAPE) package version 1.0.0 (https://github.com/WFP-VAM/modape, accessed on 22 September 2022). The algorithm implementation is based on the Whittaker–Eilers smoother exhaustively described by Eilers [74] and Atzberger and Eilers [75]. The use of specific sparse matrix routines makes the smoother fast and memory efficient. We run an optimization process to find the optimal smoothing parameter *λ*. The amount of smoothing was optimized by applying the V-curve, a variation on the L-curve. The algorithm handles missing data points easily [49] (see Section 2.1.2).

### 2.6 Experimental Setup

The LAI sample database was bounded between 3 September and 2 November 2020 seeking to prioritize the response of the LAI retrieval model within the vegetation greenness stage [63]. Additionally, 12 samples of non-vegetated surfaces were included in the field data aiming to improve the versatility of the cropland LAI retrieval model. We conducted the following strategies to find the best-fitted model for LAI retrieval based on S1 data. We first divided the tests into two groups to facilitate managing the statistics results. The first group comprises the S1 polarimetric preprocessed data without smoothing and the second set of tests involves the implementation of the optimized Whittaker smoother. We analyzed separately the performance of each S1 single-orbit data (path 141 for S1-A and path 68 for S1-A and S1-B) and then the contribution of merging multiple incidence angles for training the GPR-S1-LAI models and the result of applying it to the band stacked S1 images. Aiming to ensure more robust retrieval results and to use the collected in situ measurements to the fullest, next, a 10-fold cross-validation (CV) [76] sampling scheme was applied. CV splits the available data into *k* subsets (*k* = 10). From these *k* sub-datasets, *k*–1 sub-datasets are selected as a training dataset (51 data points, 5 on average in each sub-dataset) and a single k sub-dataset (5 data points) is used during the testing stage. The CV process is repeated iteratively *k* times [37]. The performance of the GPR-S1-LAI models was assessed using diverse goodness-of-fit metrics: the coefficient of determination (*R*^2^), the mean absolute error (*MAE*) (see Equation (17)), the root mean square error (*RMSE*) (see Equation (18)), the normalized root mean square error (*NRMSE*) (see Equation (19)), and the total time (training and validation) for the execution of the MLRA were registered. We present bellow the formulation used for *R*^2^, *MAE*, *RMSE* and *NRMSE* calculations:

(16) $$R^{2}=1-\frac{\sum_{i=1}^{N}{\left(y_{i}-{\hat{y}}_{i}\right)}^{2}}{\sum_{i=1}^{N}{\left(y_{i}-{\bar{y}}_{i}\right)}^{2}}$$

(17) $$MAE=\frac{1}{N}\overset{N}{\underset{i=1}{\sum^{\text{​}}}}\left|y_{i}-{\hat{y}}_{i}\right|$$

(18) $$RMSE=\sqrt{\frac{1}{N}\overset{N}{\underset{i=1}{\sum^{\text{​}}}}{\left(y_{i}-{\hat{y}}_{i}\right)}^{2}}$$

(19) $$\text{NRMSE}\;=\frac{\;\text{RMSE}\;}{\left(y_{\max}-y_{\min}\right)}$$

where ${\left\{y_{i}\right\}}_{i=1}^{N}$ are the *N* winter wheat LAI measured values used for model training, ${\left\{{\hat{y}}_{i}\right\}}_{i=1}^{N}$ are the LAI estimated values based on S1 polarimetric data, (*y_max_* – *y_min_*) is the in situ measurement range, and $\bar{y}$ is the mean of the LAI in situ measured values.

### 2.7 Delineation of Retrieval Workflow

**Figure 4:**
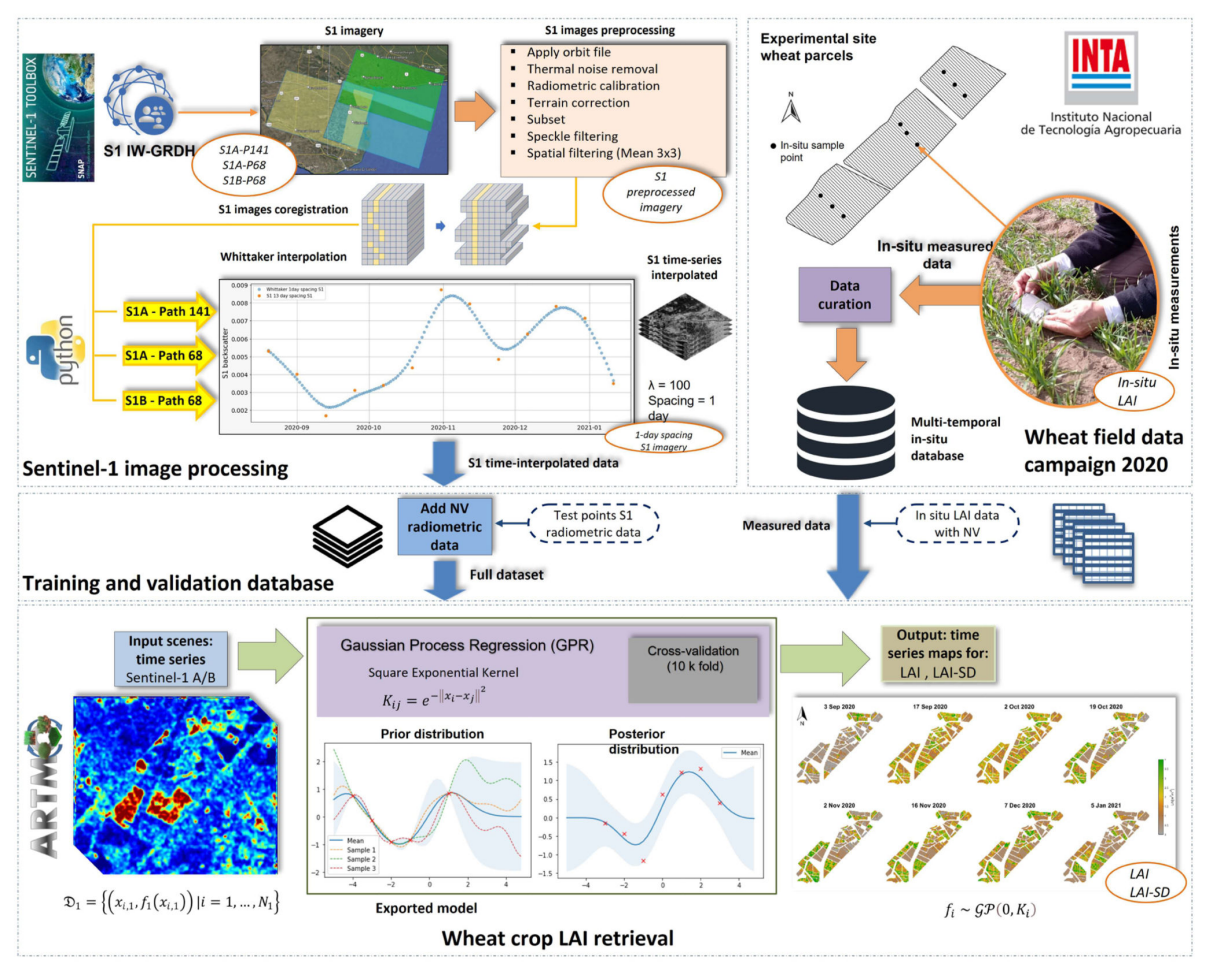
Retrieval workflow for the GPR-S1-LAI modeling using multiple local incidence angles of S1 polarimetric data, partly adapted from [63]. The output maps show our S1-derived LAI maps over the BVCR study area.

The entire S1-based retrieval workflow is visualized in Figure 4. Three well-differentiated structural blocks are detailed, starting with an S1 preprocessing section, followed by field data collection, and the probabilistic inference of LAI applying GPR models to S1 interpolated data. In brief, the implemented workflow consists of the following four main steps:

1. Pe-processing of the multiple relative orbit number S1 VH+VV polarization imagery: S1-A path 141, 68 and S1-B path 68;
2. Building the in situ database containing multitemporal field LAI measurements from the BVCR site and S1 post-processed interpolated polarimetric data,
3. Training S1 data with GPR algorithms and applying the retrieval model to obtain LAI;
4. seasonal mapping of LAI over irrigated winter wheat fields and corresponding uncertainties using the GPR-S1-LAI model.

The GPR processing was entirely operated within the Automated Radiative Transfer Model Operator (ARTMO) toolbox [77]. ARTMO was developed as a modular graphical user interface in Matlab, to automate the simulation of Radiative Transfer Modeling [40] and mapping applications. More information can be found at: http://artmotoolbox.com/ (accessed 20 September 2022).

## 3 Results

### 3.1 Optimized S1 Stack Selection for LAI Modeling

**Figure 5:**
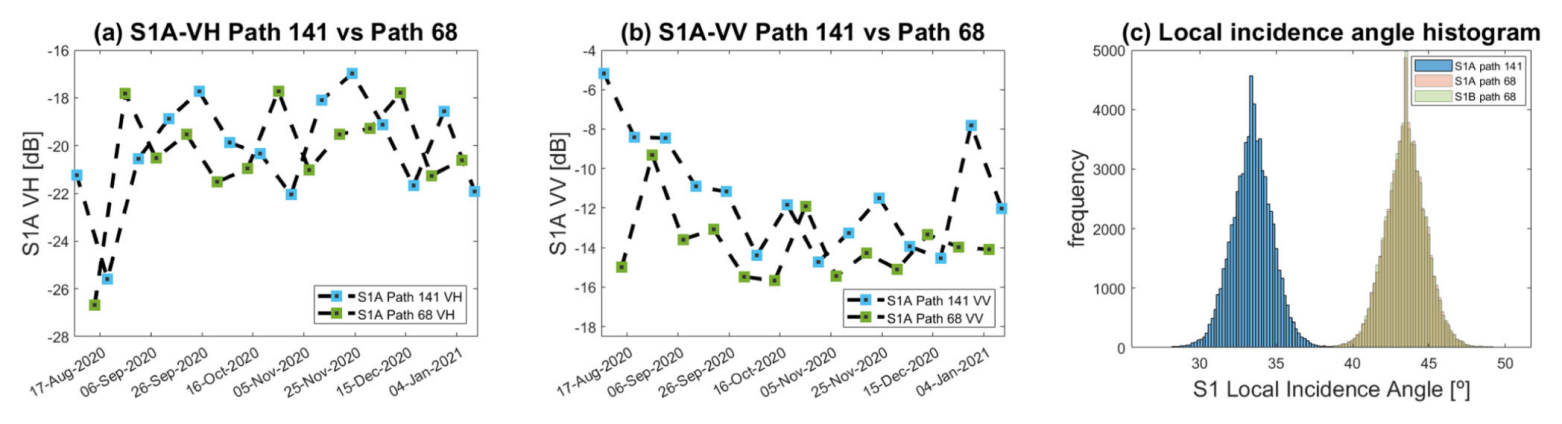
S1–A acquisition trends over the study site. (**a**) Time-series of S1–A VH polarization data for path 141 against path 68. (**b**) Time-series of S1–A VV polarization data for path 141 against path 68. (**c**) S1 Local incidence angle histograms.

We first explored the contribution of the S1 SAR data at distinct local incidence angles and its effect on the LAI retrieval models’ statistics. To do so, the S1 backscatter response for the winter wheat cropland was analyzed by plotting the VV and VH time series of polarimetric data for the distinct S1 local incidences angles. Figure 5a,b displays the S1 VH and VV trend along the crop’s phenological development. A noticeable difference can be appreciated when the S1-A path 141 SAR data is plotted against path 68 for both polarizations. Besides the lack of temporal synchronicity between S1-A acquisitions, an amplitude offset can be attributed to the differences in the local incidence angle of both S1 paths. Figure 5c, shows the S1 local incidence angle histograms for all winter wheat pixels at the study site. There is a glaring overlap between S1-A and S1-B LIA values for path 68, this results from the fact that both satellites share the same relative orbit number. The mean LIA value for S1 path 141 is around 33° whereas for path 68 it is approximately 43°.

Table 3 presents the regression statistics when the S1 original data are used to train the retrieval models in contrast with the case in which the optimized Whittaker smoother is applied to the data. It can be noted that the increase of R^2^ and the decrease of MAE, RMSE, and NRMSE values in general terms when all S1 acquisitions at distinct local incidence angles are used to train the LAI retrieval models.

**Figure 6:**
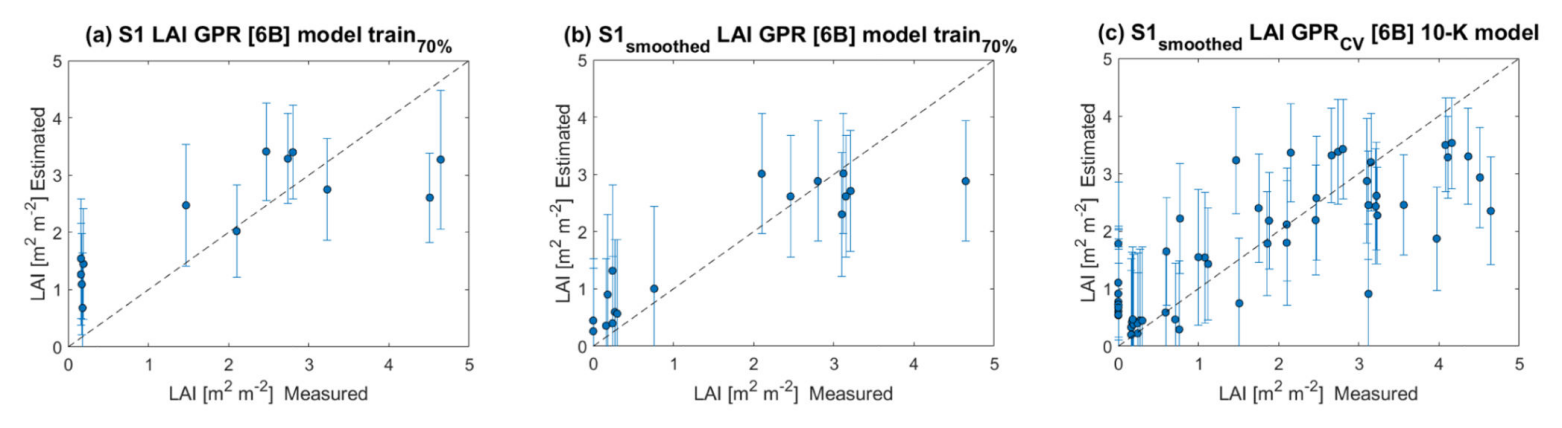
Measured vs. estimated winter wheat LAI along 1:1–line, including uncertainty intervals. (**a**) LAI model estimates using multiple local incidence angles of S1 non-smoothed time-series data; (**b**) LAI model estimates using S1 smoothed time-series data at multiple local incidence angle acquisitions and 70 % of the data for model training; (**c**) LAI model estimates using S1 smoothed time-series data at multiple local incidence angle acquisitions and 10–fold CV for model training.

Figure 6 shows the scatter plots of estimated against in situ measured LAI values. We analyzed three algorithm training scenarios. The first contemplates S1 non-smoothed data for multiple local incidence angle acquisitions (see Figure 6a). In the second, the effect of smoothing the data is studied by training the six bands (6B) GPR-S1-LAI model with 70 % of in situ samples (see Figure 6b). Finally, the third scenario investigates the contribution of a 10-fold CV strategy during the model training stage (see Figure 6c). Obtained R^2^, MAE, RMSE, and NRMSE statistics denote high agreements between retrieved and measured wheat LAI. The GPR uncertainties are also provided in the standard deviation (SD) form expressed by vertical bars.

### 3.2 Winter Wheat Seasonal LAI mapping

The 10-fold GPR_*CV*_ retrieval model was subsequently applied to the S1 stacked images for LAI mapping purposes. The S1 scenes are characterized by areas of intense agricultural usage covering a variety of crop types (see Figure 2). The wheat croplands are flat, and the vegetation growth is uniform; hence, the S1 backscatter signal is not affected by adverse acquisition geometrical effects, such as radar shadow, foreshortening, and layover. Land covers such as man-made surfaces, water bodies, or bare soil in the study region were excluded from the S1 stacked scenes by applying a vectorial mask.

**Figure 7:**
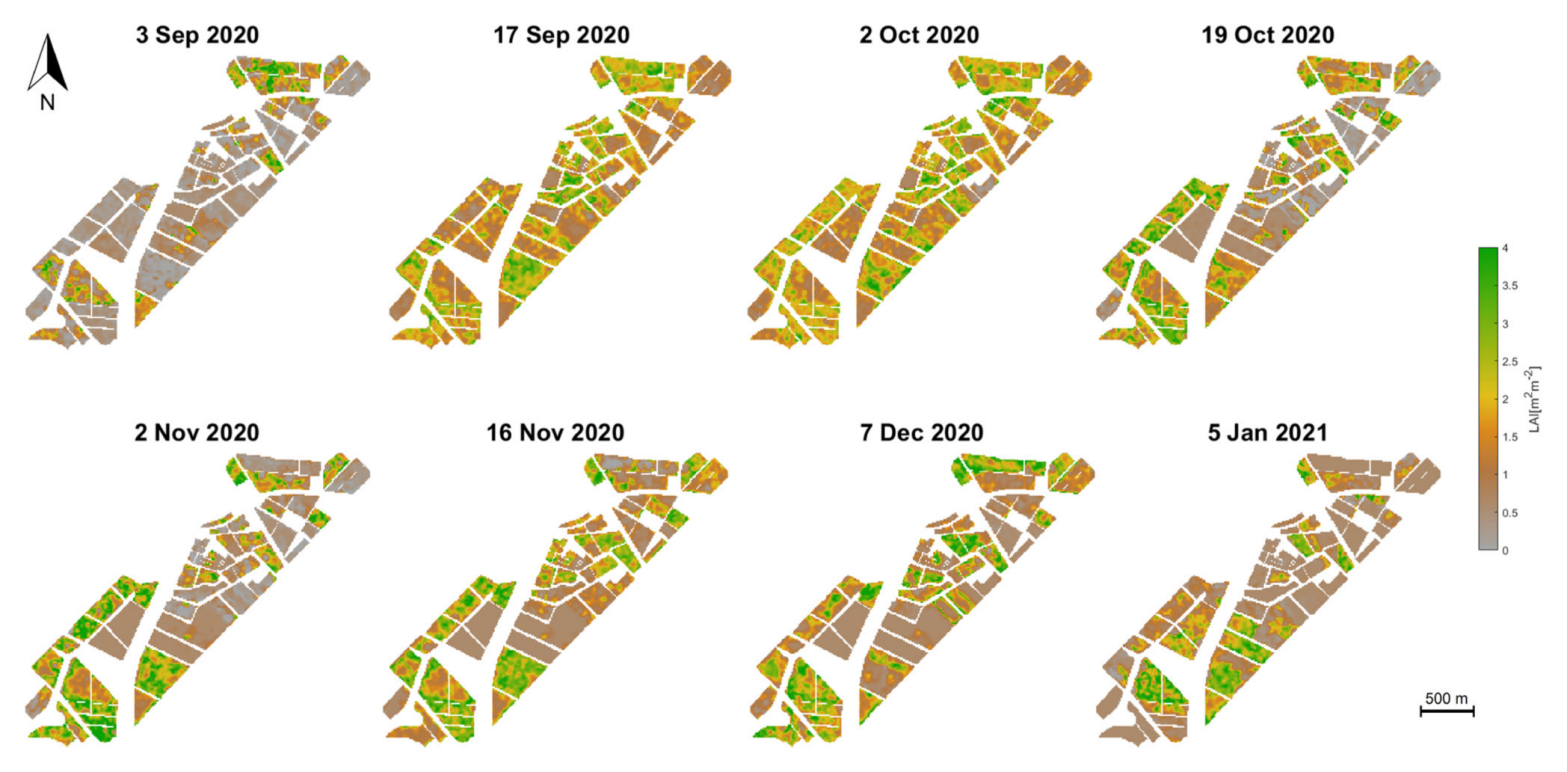
Seasonal mapping of LAI (m^2^ m^−2^) of croplands in the BVCR for the winter wheat campaign 2020, retrieved by the GPR_*CV*_ model using S1 polarimetric data from multiple local incidence angles acquisitions and in situ measured LAI data.

The eight S1-derived maps of winter wheat LAI for the BVCR 2020 campaign are shown in Figure 7. The winter wheat sown in the study site took place by the end of June 2020. The emergence of the plant stem and three tillers was noticeable in late August. By the beginning of November 2020, wheat plants are in the anthesis (flowering) stage, leading to the maximum retrieving values of LAI with 3.78 m^2^ m^−2^ on average. The senescence arrives by the middle of November 2020, while harvest takes place by the first days of January 2021.

**Figure 8:**
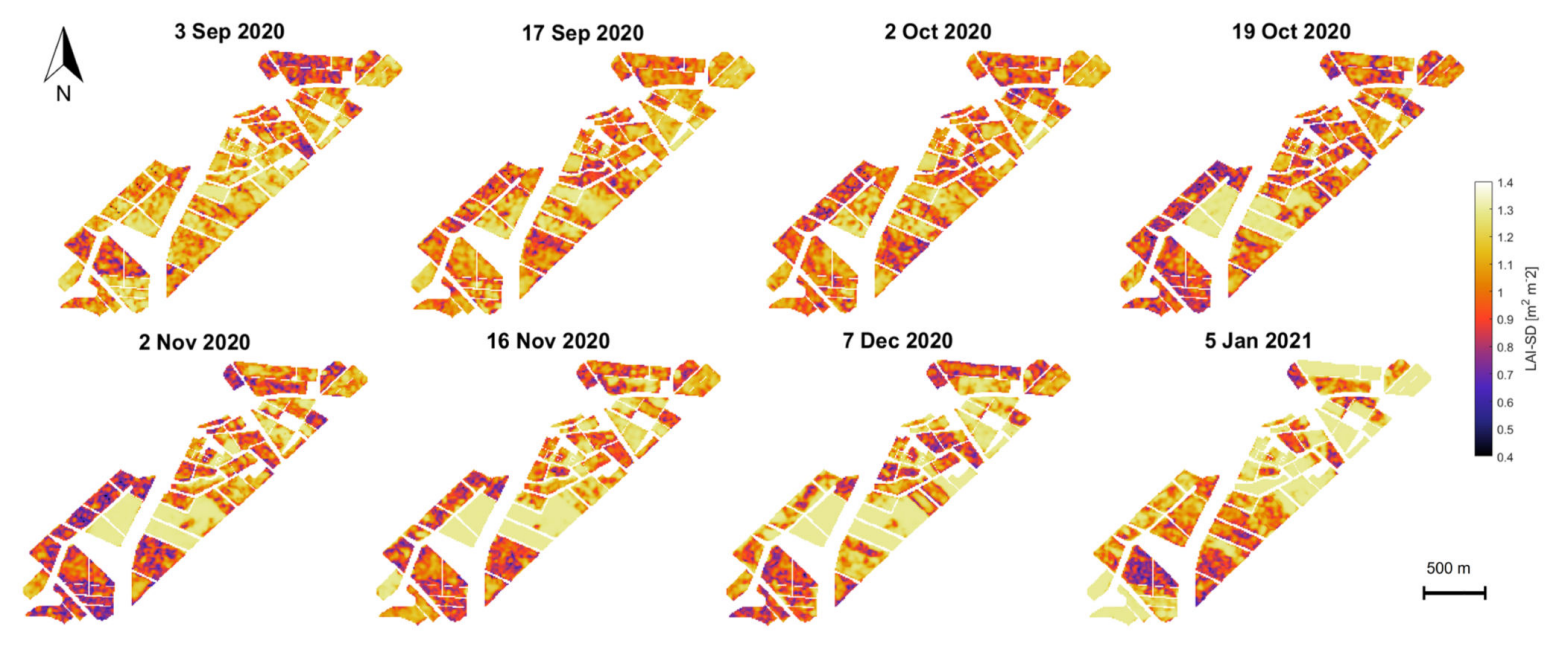
Seasonal mapping of LAI-SD (m^2^ m^−2^) of croplands in the BVCR for the winter wheat campaign 2020, retrieved by the GPR_*CV*_ model using S1 polarimetric data from multiple local incidence angles acquisitions and in situ measured LAI data.

Absolute uncertainty maps are also provided in the form of SD (see Figure 8). The associated uncertainty maps can serve as a quality layer, e.g., to exclude uncertain areas [78,79]. Generally, the GPR model produced sufficiently low LAI uncertainties (i.e., less than 0.6 m^2^ m^−2^ on average) from the start of the season to the maximum greenness, with higher values over the senescence stage, a period in which uncertainty can reach up to 1.25 m^2^ m^−2^ values.

**Figure 9:**
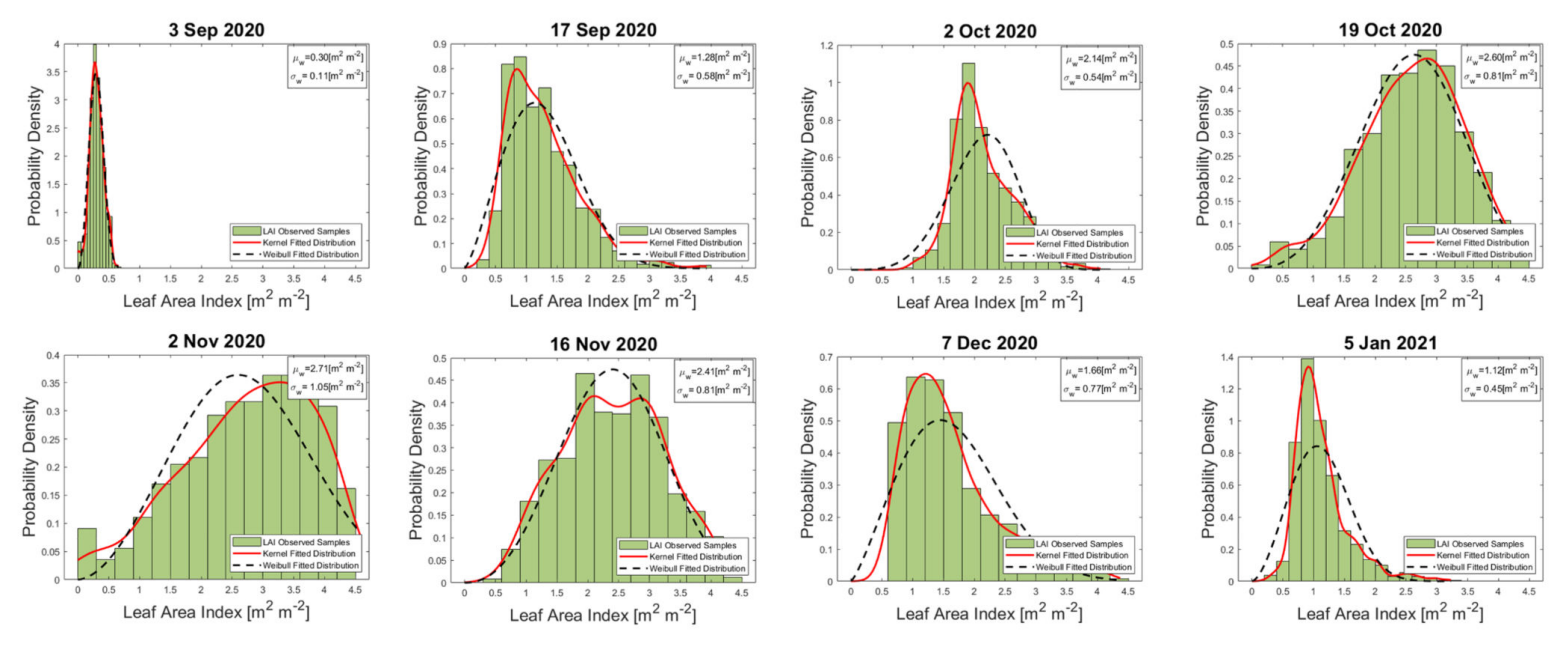
Temporal evolution of probability density function of LAI (m^2^ m^−2^) for the winter wheat paddocks 321, 323, and 323 at the study site. LAI observed samples in each graph correspond to S1-derived LAI estimations.

We computed the histograms of the retrieved LAI values for the winter wheat paddocks pursuing to visualize the dispersion of LAI values and their trend over time. On every date on which the S1-derived LAI map was obtained, we analyze the probability density function (PDF) of all pixels. We focused on paddocks 321, 322, and 323 for the BVCR 2020 field campaign. Two PDFs were explored: Weibull [80] and non-parametric Kernel probability density distribution [81]. We calculated the mean (*μ*) and the standard deviation (*σ*) values of the Weibull distribution. The results are shown in Figure 9.

### 3.3 Time-Series Trend of Retrieved LAI and Associated Uncertainty

**Figure 10:**
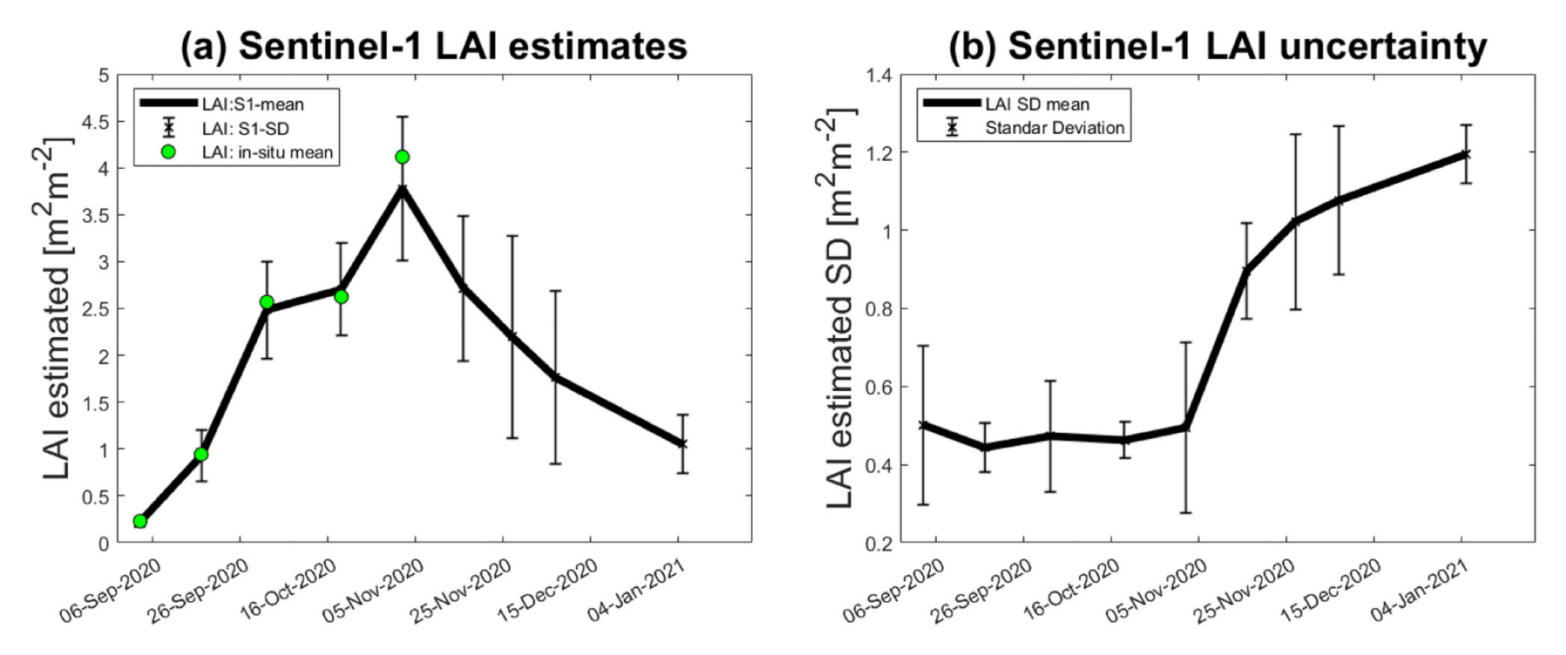
Seasonal evolution of wheat cropland over the three paddocks at the BVCR study sites described by LAI, mean values of nine ESUs within the cropland limits, and the associated uncertainty, plotted as vertical bars. (**a**) LAI estimates; (**b**) LAI uncertainty (SD).

This section explores the winter wheat’s LAI seasonal evolution along the BVCR 2020 campaign. The GPR-S1-LAI model was applied to a total of nine S1 stacked scenes with the aim of reproducing, as faithful as possible, the LAI curve of the winter wheat crop. The LAI retrieved curve evolution for the three selected winter paddock in the study region is displayed in Figure 10a. The GPR_*CV*_ model uncertainties were mapped in the form of SD to represent the variability of the LAI retrieved values (see Figure 10b). The figures show the temporal LAI and LAI-SD trends of the averaged nine ESUs (solid line), and the mean of the in situ measured LAI values (green dots). Only the first five wheat LAI measurements were plotted, excluding those samples acquired on November 16 and 30 and also 16 December 2020.

**Figure 11:**
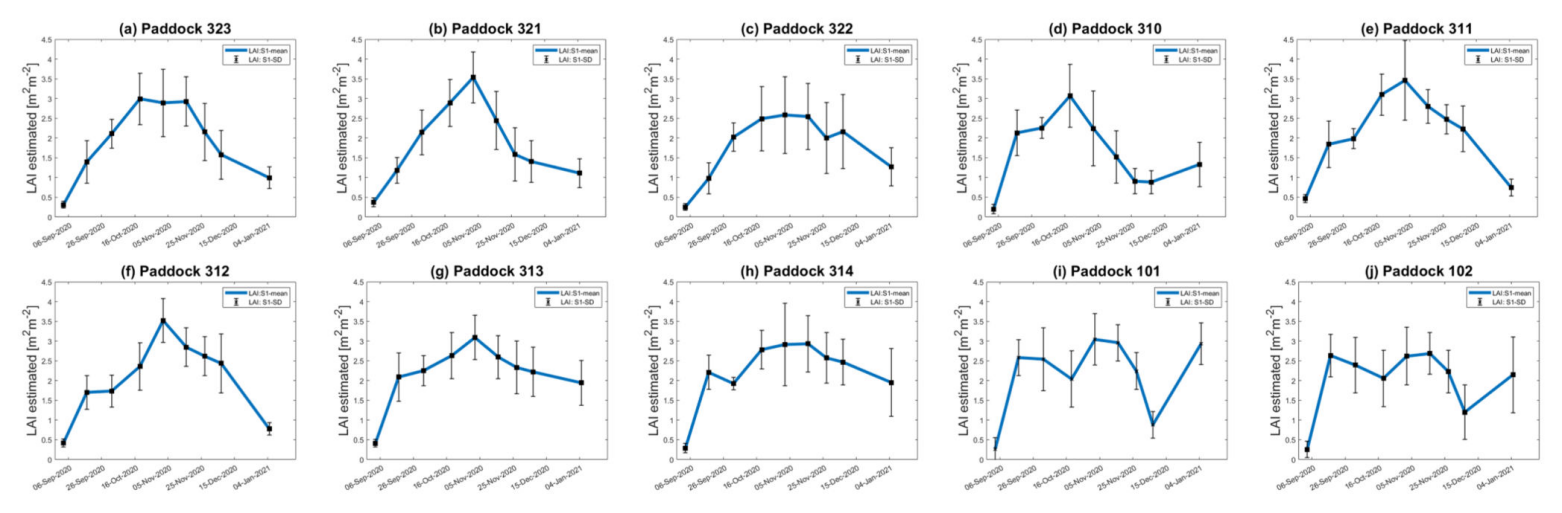
Seasonal evolution of LAI for all winter wheat paddocks in the BVCR study sites. The estimate uncertainty is represented as vertical bars. (**a**) Wheat paddock 323; (**b**) Wheat paddock 321; (**c**) Wheat paddock 322; (**d**) Wheat paddock 310; (**e**) Wheat paddock 311; (**f**) Wheat paddock 312; (**g**) Wheat paddock 313; (**h**) Wheat paddock 314; (**i**) Wheat paddock 101; (**j**) Wheat paddock 102.

For the three wheat paddocks in the study site, LAI increased from the plants’ emergence stage onwards and decreased after tillering. Abrupt drops in LAI in January 2021 suggest a harvest event. Considering the availability of the land use data for the BVCR 2020 campaign presented in Figure 2, the LAI temporal evolution of all wheat paddocks in the study site was determined by averaging all pixels inside each parcel´s bounds. Figure 11 displays the retrieved LAI temporal trend for all the winter paddocks at the study site for the BVCR 2020 campaign. Despite lacking cropland management information for all winter wheat paddocks, the LAI trend over time shows consistency with the development of wheat plants in the study region’s environment (see Sections 2.2.1 and 2.2.2).

## 4 Discussion

We explored the suitability of multi-S1 data for all-weather LAI wheat monitoring. We aimed to build a radar-based retrieval workflow based on multiple S1-A and S1-B acquisitions at distinct local incidence angles and in situ measurements of irrigated winter wheat LAI from the BVCR 2020 campaign. In the following, LAI retrieval performance and uncertainties (Section 4.1), sensitivity of S1 backscatter to winter wheat LAI (Section 4.2), the role of S1 acquisition geometry (Section 4.3), and finally the potential of seasonal trends identification based on S1 polarimetric data for wheat agronomic management (Section 4.4) are discussed.

### 4.1 LAI Retrieval Performance and Uncertainties

In terms of local incidence angles, regressions parameters at 43° (path 68) outperformed those obtained at 33° (path 141). This is in line with the vegetation-acquisition geometry dependency of the radar-acquired scenes (see Figure 5 and Table 3). When both descendent orbits (141 and 68) were used to train the GPR-S1-LAI retrieval model, an appreciable R^2^ increase occurred compared to the use of single-orbit polarimetric data. This can be attributed to additional information on the vegetation structure of the different dates and from distinct angles of observation. This achievement is remarkable because mapping winter wheat LAI using multiple S1 observations at distinct local incidence angles has not yet been evaluated so far. We found a significant improvement in the R^2^ and RMSE of the GPR-S1-LAI model after applying the data smoothing process based on the optimized Whittaker smoother (see Table 3).

Based on the information presented in Table 3, we can conclude that the best results were obtained for model *S1-AB-P141-68*, with NRMSE < 15 % and R^2^ of 0.85 m^2^ m^−2^. Nevertheless, and despite achieving these high-accuracy retrieval parameters, we focused on the CV model, seeking to fully exploit the training dataset. The CV strategy provided greater robustness to the LAI retrieval model, while preserving relatively high accuracy. The GPR_*CV*_-based LAI model of winter wheat yielded a relatively high R^2^ with 0.67 and a low RMSE of 0.88 m^2^ m^−2^.

The LAI estimates based on our approach suggested superior performance over those presented in previous studies. For instance, Bousbih et al. [17] analyzed the potential of S1 radar data for retrieving LAI of cereals in agricultural areas over the Kairouan Plain (Tunisia, North Africa). They achieved R^2^ = 0.25 using dual polarimetric radar data acquired from a single angle of observation. Hosseini et al. [51] used full-polarimetric (HH+HV+VH+VV) RADARSAT-2 data for soybean and corn LAI mapping achieving R^2^ = 0.64 RMSE = 0.63 m^2^ m^−2^ and R^2^ = 0.66 and RMSE = 0.75 m^2^ m^−2^, respectively.

Regarding the LAI retrieval uncertainties, two issues deserve to be adequately addressed. First, S1 SAR images are conditioned by the radar´s inherent speckle noise, which affects the VH and VV backscatter amplitude of adjacent pixels of homogeneous monitored cropland. This speckle noise, mainly due to the relative phase of individual scatters within a resolution cell, increases the uncertainty of the LAI estimates. Consequently, the S1-based LAI values have high dispersion between retrieving dates (see Figure 9). When a particular scatter element is observed at distinct local incidence angles repeatedly on time, this random speckle effect can be mitigated. Multiple radar observations increase the amount of information, minimizing the entropy of the data and the uncertainty of the estimates. Considering world locations where ascending and descending orbits of S1 are both available, the approach presented in this study constitutes an auspicious line of research. Secondly, the in situ measurements (see Table 1) were collected during the wheat growing season, which renders the models more robust and confident. It can be noticed that in situ LAI sample values show a different pattern than the SAR LAI estimates in late November and December 2020 (see Figure10), while field trait data show high LAI values, S1-based retrieved LAI show a reduction during this timeframe. After the anthesis, the photosynthetic activity of winter wheat plants decreases leading to the beginning of the senescence process. The moisture content of wheat plant stems, leaves, and ears is considerably reduced by this time affecting the radar backscatter. The in situ measured database was then restricted between 3 September to 2 November 2020 seeking to preserve the consistency of the LAI retrieved values along the green vegetative stages of the winter wheat crop. The final GPR-S1-LAI model is therefore better adapted toward green vegetation states. Hence, the uncertainty of the developed models will increase during later (mature/senescent) growing stages.

### 4.2 Sensitivity of S1 Backscatter to Winter Wheat LAI

LAI can be differentiated into LAI green and LAI brown. The green leaf area index (LAI_*G*_) represents the photosynthetically active leaves of the plants, and is thus also the most common type of LAI [82]. The brown leaf area index (LAI_*B*_), represents the normalized leaf area, which is senescent and losing photosynthetic function [79,83]. During the greenness stage, wheat plant development is usually modeled by LAI_*G*_. From the first days of August to 16 November 2020, winter wheat plants remain green. During this period, LAI_*G*_ increased as the wheat plants grow. By 16 November 2020, crop senescence started, and the share of brown leaves increased. From this point on, the LAI_*G*_ starts decreasing constantly until reaches zero value at the harvest date. Consequently, the S1 backscatter at VH and VV polarization remained stable at low values (see Figure 5). Additionally, by 16 December 2020, the wheat heads became completely dry, thus, the C-band backscatter decreases to reach the lower monitored values at this stage, the vegetation becomes transparent to the radar signal and the soil moisture is very low because irrigation is stopped. These structural changes in the canopy lead to an increased volume scattering of the ground targets that can be mainly distinguished at VH polarization. Mattia et al. [20] and Satalino et al. [84] found that when the heads of wheat plants emerged, the backscatter changed drastically. They also highlighted the importance of the heading stage as a turning point at which the C-band radar backscatter becomes essentially sensitive to soil moisture rather than wheat plants’ biomass variations. During the senescence phase (from the greenness maximum until wheat plants have completed the dehydration process), LAI measurements are indeed represented by the total LAI (LAI_*T*_), thus LAI_*T*_ = LAI_*G*_ + LAI_*B*_, implying that the LAI is defined by green and brown leaf structures [79,83]. The splitting of the analysis into two periods (greenness and senescence) was studied by Che et al. [85], who analyzed the temporal trend of the LAI of vegetation in Shandong Province, China. They set up a breakpoint of two curves (LAI_*G*_ and LAIB) that occurs during the flowering period (anthesis) when the wheat has reached its full height. As radar is sensitive to the canopy’s moisture and structure, it implies a certain sensitivity to LAI_*T*_. Nasrallah et al. [24] studied the temporal profiles of the widely known Normalized Difference Vegetation Index (NVDI) based on Sentinel-2 optical data and the S1 backscatter at VH and VV polarizations. The authors remarked on the high sensitivity of the SAR signal to winter wheat phenological cycle, in comparison to the time-series of NDVI [27].

### 4.3 Role of S1 Acquisition Geometry

The LAI temporal trend for all winter wheat paddocks in the study site is presented in Figure 11. Even though winter wheat croplands are typically homogeneously distributed, the spatial orientation of the rows in the wheat paddocks was not uniform between the cropland locations. For example, in the three selected winter wheat paddocks for in situ data collection (paddocks 321, 322, and 323), the rows were oriented from west-southwest to east-northeast, while in paddocks 101 and 102, the orientation was from north-northwest to south-southeast. In addition, the rows for paddocks 311–314 were from west to east, while in paddock 310, the rows were oriented from west-southwest to east-northeast. This difference in the spatial orientation of rows can be more noticeable during the first development stages of winter wheat plants, such as seedling growth and tillering, when the soil is not yet fully covered by vegetation. The S1 radar signal during this period is governed by the soil moisture dynamic, which differs from one winter wheat paddock to another, giving the difference in soil irrigation conditions and surface roughness. The S1 C-band radar backscatter was affected by the spatial orientation of the rows which varied for the winter wheat paddocks in the study region. This is based on the dependency of the target’s radar cross-section (RCS) on the satellite’s relative angle [22], thus even minimal changes in the target aspect significantly affect the RCS [18] (see Figure 5). The descending orbits 141 and 68 were used to perform early morning measurements when dew may become a confounding factor [11,35]. During morning hours of the S1 acquisition, the cropland relative humidity is high and the soil moisture increases causing backscatter alterations of the SAR signal [86,87]. Fortunately, the S1 overpass time was similar for both orbits (around 6:20 h local time), leading to a comparable influence of the dew effect for all S1 acquisitions. After heading had occurred, the soil contribution is considerably reduced and the volumetric backscattering of the vegetation’s canopy became more significant at 43°. This finding was noted by several previous studies [20,26,88,89].

Inconsistencies between the trend of the LAI in Figure 11 can be noticed for winter wheat paddocks 101, 102, and most importantly, in paddock 310 (peak LAI is end-of-October and then decreases) and the in situ measurements (highest LAI values were observed throughout November and December). These differences may be attributed to the fact that each crop parcel has been managed by different farmers at the study site regardless of the scope of this research. Other factors accounting for this issue are crop row orientation, the inherent SAR speckle noise, and the tri-dimensional plant structure of winter wheat detected by the S1 radar instrument. In addition, wheat paddocks 101 and 102 were harvested on 18 December 2020, while 321, 322, and 323 on 4 January 2021. Ultimately, the S1-derived LAI decreases suddenly for all winter wheat paddocks between 18 December 2020 and 5 January 2021, which is due to the harvest period. After these dates, there is an increase in LAI values in January for paddocks 101, 102, and 310, which is due to post-harvest land management practices. The sensitivity to surface soil moisture and roughness also increases by this time.

### 4.4 Potential of Seasonal Trend Identification Based on S1 Polarimetric Data for Wheat Agronomic Management

S1-based LAI mapping represents an attractive all-weather strategy for space-based monitoring of croplands, particularly in cloudy agriculture areas such as the BVCR. Characteristics of the study region were addressed in Section 2.2.1 and the irrigated winter wheat development and properties in Section 2.2.2. This valuable information can support an agronomic analysis of the winter wheat phenology in the study region. A summary of the most important points concerning the previous sections (see Sections 2.2.1, 2.2.2, and 2.3.1), and the innovative S1-based LAI mapping model is given next.

The LAI response was evaluated for a typical wheat extensive crop condition at a productive scale at the BVRC. Agronomic nutritional and water requirement management was performed to enable maximum yield potential. We can appreciate the development of a typical phenological curve concerning each of the sampling dates. A positive evolution was observed in LAI measurements in response to fertilizer and irrigation applications during the vegetative stage of the crop (first five sampling dates). The registered precipitation at the study site along the crop phenological cycle was representative of the expected averaged value of the BVCR semi-arid region (see Section 2.2.1).

In Figure 10a, a noticeable decline in LAI evolution from 5 November (3.78 m^2^ m^−2^) to 25 November 2020 (2.65 m^2^ m^−2^) was observed. This coincides with the stages of the ontogenetic cycle of the wheat crop at the study site and with the warm temperatures recorded for those dates (see Figure 3), thus accelerating the rate of crop development and promoting flowering. In addition, as the photoperiod increases in November in the southern hemisphere, crop stages are shorter [90]. The observed decline is explained by the crop cycle interchange related to changing the apex to the reproductive stage and the beginning of spikelet differentiation [91]. The onset of tillering occurs after the appearance of the terminal spikelet at the apex. The beginning of internode elongation determines a change in assimilate partitioning within the plant, which is mainly destined for the growth of the stem, and consequently, the production of tillers ceases. From that moment on, and depending on the available resources, tiller mortality will occur, defining at the end of this process the number of spikelets per unit area [92]. Therefore, it is possible to make an analogy between the beginning of elongation and the moment when the estimated S1-derived LAI decreases similar to the results obtained by other authors [24,27]. This evidence confirms the capability of S1 radar data to detect changes that are diagnostic of wheat crop phenology [93], especially over cloud-prone agriculture areas.

## 5 Conclusions

Merging distinct incidence angle observations of SAR potentially provides a richer source of information related to vegetation structure than single narrow observations, and can thus efficiently support all-weather cropland monitoring applications. Here, we presented an S1-based retrieval workflow for operational mapping of LAI optimized for an irrigated winter wheat cropland located in the South of Buenos Aires Province in Argentina. The implemented retrieval method used the advantages of integrating two distinct S1 descending relative orbits. Physical interaction between the radar signal at distinct acquisition geometries and the vegetation structure provided complementary information for LAI retrieval. We chose GPR as a solid probabilistic MLRA for the retrieval of LAI, given the advantage of delivering associated uncertainties along with the estimates, so assisting in the reliability assessment of the LAI retrieval.

The GPR_*CV*_ model for retrieving LAI over winter wheat was validated with relatively high accuracy against in situ data RMSE = 0.88 m^2^ m^−2^ and R^2^ = 0.67. The established GPR_*CV*_ LAI model was posteriorly applied to a series of S1 stacked imagery of the growing season of 2020 over the BVCR study site. The resulting maps suggest spatiotemporal consistency with winter wheat growth in the region, however, the transferability of the retrieval model to other cropland environments remains to be carefully analyzed. We conclude that dense S1 time-series data at both ascending and descending orbits present an appealing opportunity for year-round monitoring of cultivated areas. Additional research is required to assess if this workflow is applicable to other vegetation structures and environmental conditions.

## Figures and Tables

**Table 1:** LAI in situ measured database.

Wheat Variable	Sampling Date	Range	Mean	SD
**LAI** (*m*^2^ *m*^−2^)	3-Sep-20	0.16–0.30	0.23	0.05
	17-Sep-20	0.56–1.54	0.94	0.29
	2-Oct-20	1.59–3.81	2.57	0.66
	19-Oct-20	1.53–3.27	2.62	0.51
	2-Nov-20	2.78–5.05	4.12	0.63
	16-Nov-20	3.31–5.39	4.02	0.80
	30-Nov-20	3.29–4.75	4.08	0.50
	16-Dec-20	3.97–5.64	4.68	0.43

**Table 2:** Field campaign and Sentinel-1 acquisition dates.

Sampling date	S1-A Path 141	S1-A Path 68	S1-B Path 68	Δ Days (AVG)
3-Sep-20	1-Sep-20	8-Sep-20	2-Sep-20	3
17-Sep-20	13-Sep-20	20-Sep-20	26-Sep-20	5
2-Oct-20	7-Oct-20	2-Oct-20	8-Oct-20	4
19-Oct-20	19-Oct-20	14-Oct-20	20-Oct-20	2
2-Nov-20	31-Oct-20	7-Nov-20	1-Nov-20	3
16-Nov-20	12-Nov-20	19-Nov-20	13-Nov-20	3

**Table 3:** Regression statistics for winter wheat LAI retrieval modeling using S1 time-series data.

Regression Statistics for Winter Wheat LAI Retrieval Models Using S1 Time-Series Data						
S1 Data	MLRA	MAE[m^2^ m^−2^]	RMSE[m^2^ m^−2^]	NRMSE[%]	R^2^	Time [s]
S1-A-P141	GPR[2B]	1.33	1.48	33.02	0.15	0.1219
S1-A-P68	GPR[2B]	1.16	1.38	30.74	0.34	0.0662
S1-B-P68	GPR[2B]	1.22	1.45	32.30	0.44	0.0893
S1-AB-P141-68	GPR[6B]	0.93	1.04	23.24	0.68	0.1305
**Regression statistics for winter wheat LAI retrieval models using S1 time-series smoothed data**						
S1-A-P141	GPR[2B]	1.10	1.27	27.28	0.38	0.2638
S1-A-P68	GPR[2B]	0.63	0.86	18.60	0.67	0.0756
S1-B-P68	GPR[2B]	1.20	1.40	30.20	0.13	0.1247
S1-AB-P141-68	GPR[6B]	0.50	0.66	14.21	0.85	0.1525
S1-AB-P141-68	GPR_*CV*_[6B]	0.68	0.88	18.91	0.67	0.0117

## Data Availability

Not applicable.
